# Value-Added Utilization of Wheat Straw: From Cellulose and Cellulose Nanofiber to All-Cellulose Nanocomposite Film

**DOI:** 10.3390/membranes12050475

**Published:** 2022-04-28

**Authors:** Hongxia Bian, Yanyan Yang, Peng Tu, Jonathan Y. Chen

**Affiliations:** 1 College of Science, Gansu Agricultural University, Lanzhou 730070, China; bhxia790311@gsau.edu.cn (H.B.); yyy15101331175@163.com (Y.Y.); 2 School of Human Ecology, The University of Texas at Austin, Austin, TX 78712, USA

**Keywords:** wheat straw, cellulose, nanocomposites, reinforcements, mechanical properties

## Abstract

To accelerate the high value-added usage of agricultural residue, cellulose and cellulose nanofibers (CNFs) were extracted from wheat straw and then formed into all-cellulose nanocomposite films. The acid–alkali method (AM) and the extraction method (EM) were respectively adopted to prepare wheat straw cellulose (WSC), and the TEMPO oxidation method was used to extract CNFs. The nanocomposite films were fabricated by dissolving WSC and adding different CNF contents of 0.0, 0.5, 1.5, and 3.0%. There was a better miscibility for the all-cellulose nanocomposite film prepared by EM (Composite-E) compared to that for the all-cellulose nanocomposite film prepared by AM (Composite-A). Composite-E also showed a better optical transparency than Composite-A. The thermal stability of the two RWSCs presented contrary results when the CNFs were added, indicating a higher thermal stability for Composite-E than for Composite-A. This should have determined the properties of the films in which Cellulose I and Cellulose II coexisted for the all-cellulose nanocomposite films, and the forming mechanism of Cellulose II and crystallinity were determined by the cellulose-extracting method. X-ray diffraction (XRD) and Fourier-transform infrared (FT-IR) spectroscopy also showed that there was more Cellulose I in Composite-E than in Composite-A. The results are expected to enrich the data for deep processing of agricultural residues.

## 1. Introduction

There were 134.25 million tons of wheat produced in China during 2020–2021 [1], which provided food for people, but also generated a large amount of agricultural waste—wheat straw. Landfills, animal feedstock, mushroom-cultivation base material, biofuel, and activated carbon are the usual ways to utilize these wheat stalks [2,3]. Although these ways enhanced the added value of the wheat straw, the broader and higher value still needed to be expanded. Wheat straw contains 35–45% cellulose, so it is an advantageous path to extract cellulose and nanocellulose from wheat straw, and to develop novel functional polymer composites for deep and high value-added utilization.

Cellulose, as the most abundant biodegradable, renewable, and biologically compatible polymer on Earth, has been widely used in textiles, paper making, plastics, food, daily cosmetics, biomedicines, water retention, oilfield chemistry, and so on in the form of cellulose and its derivatives [4]. Cellulose is a main form for many end uses and has been applied in a wide range of materials and products for thousands of years [5], and is still one of the most attractive and potential materials used to date. In addition, natural cellulose consists of nanoscale fibrils and crystallites [6]. Nanocellulose not only has the basic structure and properties of cellulose, but also has the characteristics of nanoparticles, such as a large specific surface area, high crystallinity, high strength, high Young’s modulus, etc. It has a natural difference with ordinary cellulose, so nanocellulose is used in more specialized industries, such as enzyme immobilization, catalysis, biosensing, sewage treatment, composites, etc. 

Crop straw is mainly composed of cellulose, hemicellulose, lignin, and small amounts of other inorganic and organic materials. Among these, cellulose is strongly enclosed by lignin and hemicelluloses in the cell wall, and its performance is affected by organic substances [7,8]. The chemical process is a common method to separate cellulose, especially the acid–alkaline method [9,10], which is based on the advantages of convenient operation and easy control. In separating, it is also one of key factor to remove organic substances, such as lipids, waxes, etc., and can improve the purity of cellulose [11]. Of course, physical methods often are applied in the extraction process of cellulose to improve the yield and efficiency. In producing nanostructured cellulose, one of the common methods is acid hydrolysis, in which the cellulose nanostructure is produced because hydrogen ions attack β-1, 4-glucosidic bonds in the cellulose chain and destroy amorphous areas. When sulfonate groups replace the hydroxyl groups in cellulose crystals in sulfuric acid hydrolysis, the thermal stability of the nanocellulose is reduced [12]. TEMPO oxidization is another method to isolate the cellulose nanostructure, and it can induce deconstruction by 2, 2, 6, 6-tetramethylpiperidine-1-oxylradical (TEMPO) oxidation. The hydroxyl groups at the C_6_ position on the cellulose surface are oxidized by TEMPO and form C_6_ carboxylate groups, which can maintain the original crystalline structure without changes and give the nanocellulose a better dispersibility and stability [13,14,15]. 

Prepared cellulose and nanostructured cellulose have led to new forms of biopolymer composites. In particular, nanostructured cellulose was used as a filler material in chemical polymers to enhance the mechanical properties [16,17,18] or to provide some functional advantages [19,20]. In 2004, Nishino first introduced the concept of an all-cellulose nanocomposite based on self-reinforcement cellulose composites [21]. Nanostructured cellulose also has been added into regenerated cellulose to fabricate various types of green composites [22,23,24,25,26]; i.e., a cellulose matrix strengthened by a cellulose nanofiber. The problem of interface adhesion is a primary disadvantage in preparing composites with two chemically foreign components [27,28]. When a composite is produced by materials with similar properties, better interface adhesion and mechanical properties can be expected. Hai fabricated an all-cellulose nanocomposite by adding cellulose nanofiber (CNF) that was isolated from deinked copy/printing paper into a cellulose matrix, and discussed the effects of a heat treatment on the composite’s properties [26]. Moein extracted cellulose from bagasse and used a disk grinder to prepare nanofibers to produce a cellulose nanocomposite film for food packing [24]. Zhao used microcrystalline cellulose (MCC) and CNF extracted from MCC to produce an all-cellulose nanocomposite [22]. These all-cellulose nanocomposite films all had better properties than cellulose film. Although it has been well known that all-cellulose nanocomposites have excellent performances due to reinforcing cellulose with native CNFs, it has not been reported that cellulose and CNFs were both extracted from the same agricultural residue and used for fabricating high value-added all-cellulose nanocomposites. 

Aiming at improving the high value-added usage of wheat straw, this study focused on a hybrid approach to extract wheat straw cellulose (WSC) and CNF to produce CNF-amalgamated all-cellulose nanocomposite films. In the processing study, WSC was prepared by two extracting methods in order to compare the property differences and effects of using the two types of cellulose as a matrix for nanocomposite films. CNF was isolated by the TEMPO oxidization method. The nanocomposite film was produced by dissolving WSC in a DMAc/LiCl solvent system, adding CNFs into dissolved cellulose, and then casting. In anticipation of the future applications using WSC, CNF, and all-cellulose nanocomposite films, the morphologies, thermal stabilities, crystal characteristics and tensile properties, and the key factors that influenced the produced film’s properties were also analyzed. A hypothesis of this study was that each step involved in the extraction of the wheat straw cellulose and nanocomposite cellulose film could be adjusted individually to enhance the end-use product’s properties. It is expected that this study can provide practical experience for other researchers and enrich the experimental data for deep processing of wheat straw and agricultural residue. 

## 2. Materials and Methods

### 2.1. Materials

Wheat straw was harvested from Qingyang, Gansu, China, and dried naturally after removing leaf sheaths. The following reagents were used to isolate wheat straw CNFs: dimethyl sulfoxide (DMSO) purchased from Shanghai Zhongqin Chemical Reagent Co. LTD, Shanghai, China; and sodium bromide (NaBr 99%), 2,2,6,6-tetramethylpiperidine-l-oxylradical (TEMPO 98%), sodium hypochlorite (NaClO 13%), and sodium sulfite (Na_2_SO_3_) purchased from Shanghai Titan Technology Co. LTD, Shanghai, China. 

The main reagents used to extract the WSC were as follows. Sodium chlorite (NaClO_2_ 84%), methylbenzene (C_7_H_8_), hydrochloric acid (HCl 37%), and sodium hydroxide anhydrous (NaOH 98%), all purchased from Shanghai Titan Technology Co. LTD, Shanghai, China. Acid-alkaliN, N-Dimethylacetamide anhydrous (DMAc 99.8%), and lithium chloride anhydrous (LiCl 98.0%) were purchased from Shanghai Zhanyun Chemical Industry Co. LTD, Shanghai, China and were used to dissolve the WSC. All of the above reagents were used as received.

### 2.2. Methods

#### 2.2.1. Preparation of CNF

The preparation method for the CNF was that referred to by Isogai [29]. In brief, we weighed 10 g of wheat straw after cleaning and drying that was soaked in 13% NaOH solution with bath ratio of 1:15 for 24 h. After cleaning by deionized (DI) water and drying, the wheat straw was soaked in a mixture of 2% NaOH and 1.5% Na_2_SO_3_ solution with a bath ratio 1:10 at 100 °C for 3 h. After DI water washing, drying, and grinding, the WSC power was obtained by sifting with an 80-mesh sieve. 

The WSC powder was immersed in DMSO at a bath ratio of 1:30, stirred for 5 h, and then cleaned with DI water before drying. The dried powder was soaked in DI water at a bath ratio of 1:100 and stirred after adding 0.03% TEMPO and 0.6% NaBr. After that, the mixture was kept at 4 °C for 12 h. The 0.4% NaClO was added into the mixture to start the reaction. To keep the pH value at 10.5, 0.1 mol/L of HCl was added. After stirring for 2 h, 5 mL of ethyl alcohol was added to stop the reaction, and the pH value was adjusted to 7 by using 0.1 mol/L of NaOH. A WSC/CNF suspension was then obtained. 

A high-speed centrifuge at 7000 r/min was used for 5 min to remove liquid in the suspension, and repeated 3–5 times to obtain the precipitate containing WSC and CNFs. After pouring the precipitate into 4 °C DI water at a bath ratio of 1:100, a high-speed homogenizer was used to disintegrate for 10 min at 15,000 rpm to obtain the CNF suspension. CNFs were obtained by freeze-drying at −50 °C. 

#### 2.2.2. Preparation of WSC 

**Figure 1:**
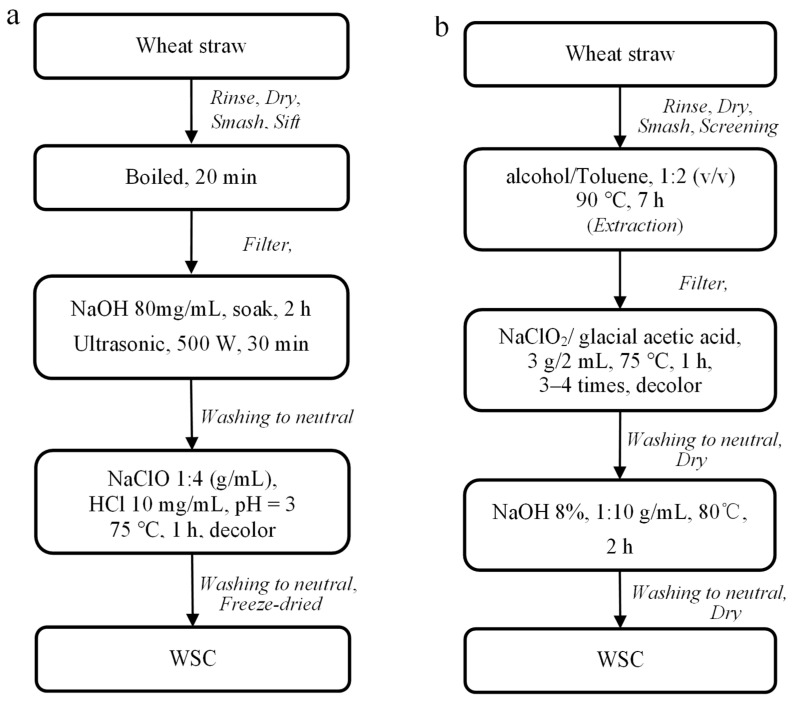
Flowchart of WSC preparation method: (**a**) AM; (**b**) EM.

The WSC was prepared according to [30], and as shown in Figure 1a. In this approach, a NaOH solution was used to remove the hemicellulose, and in an acid condition, the lignin was removed. This method is denoted as the acid–alkali method (AM) in this paper. As a second approach, as shown in Figure 1b, the WSC was prepared according to previously reported research [31]. In this approach, cellulose was pretreated by removing fats, waxes, and other impurities using a Soxhlet extractor. This method is termed as the extraction method (EM) in this paper.

#### 2.2.3. Fabrication of All-Cellulose Nanocomposites

Since all-cellulose film has many promising applications, such as in packaging, medical dressing, mulching, films, and so on [19,20,24,27], the cellulose and CNFs from wheat straw were used to prepare an all-cellulose film. Following a previous work [32], 3 g of WSC was added to 97 g of DMAc/LiCl solvent, including 6 wt% LiCl, in 100 g DMAc. After stirring for 2 h at 120 °C, the solution temperature was adjusted to 50 °C for a continuous stirring for 5 h. When the WSC was dissolved completely to form a transparent solution, the cellulose solution was casted between gaskets with a fixed thickness that were placed on a glass plate. The casting zone was a rectangular area used to form a cellulose film with dimensions of 300 mm in length, 40 mm in width, and 0.4 mm in thickness. After the WSC solution gelated in air for 12 h, it was cut into rectangular samples and put into DI water for 5 days to wash off the DMAc/LiCl solvent and to form regenerated wheat straw cellulose (RWSC). During this cellulose regeneration, the DI water was changed every day. The soaked RWSC gels were laid between two glass plates pressed with a dead weight for film formation by drying at ambient room conditions (23 °C, 45% RH). To fabricate the all-cellulose nanocomposite film, CNFs were added into dissolved cellulose at concentrations of 0.1%, 1.5%, and 3.0% after the WSC was dissolved for 30 min at 120 °C. The steps for film casting were similar to those described above. 

### 2.3. Instrumental Characterizations

#### 2.3.1. CNF and Film Morphology

A quantity of 0.5 mg of CNF was dispersed in 100 mL of DI water. A drop of suspension was placed on a glass slide to dry in air for the test. The morphology of the CNFs was measured by an atomic force microscope (AFM) (Asylum Research MFP 3D classic). The surface and cross-sectional characteristics of the RWSC and cellulose nanocomposite films were examined using an ultrahigh-resolution scanning electron microscope (SEM) (Hitachi S-5500) with operating voltage of 5.0 kV. The specimens were coated with gold before observation.

#### 2.3.2. Crystal Structure 

The crystal type and crystallinity of all the regenerated cellulose and CNF nanocomposite samples were investigated using the X-ray diffraction method (XRD) (Puxitongyun XD-2) with nickel-filtered Cu Kα radiation (λ = 0.1540 nm), an operating voltage of 40 kV, a current of 30 mA, and a scanning range of 10–45° with a step size of 0.01° min^−1^. The X-ray diffraction patterns were simulated using the following mathematical model [33,34,35]:

(1) $$T=\overset{n}{\underset{i=1}{\sum }}S_{i}+Z$$

where *T* is the total diffraction intensity, *S_i_* is the intensity of each diffraction peak, *Z* is the intensity of the amorphous diffraction, and *n* is the number of diffraction peaks. 

The following diffraction-intensity ratio was used to calculate the crystallinity index (CrI%):

(2) $$\mathrm{CrI}\;\%=\frac{S_{i}\times 100}{T}$$

in which the following peak separation method was used to determined *S_i_* [36]:

(3) $$S_{i}=f_{i}G_{i}+\left(1-f_{i}\right)L_{i}$$

where *G_i_* and *L_i_* are Gaussian and Lorentz functions, respectively, and *f_i_* is a fractional coefficient.

The crystal type of the wheat straw products was also analyzed using Fourier-transform infrared (FT-IR) spectroscopy (Thermofisher Nicolet IS 50), based on its sensitivity in detecting the cellulose polymer’s structural features. All spectra were recorded with an accumulation of 64 scans at a resolution of 4 cm^−1^, from 4000 cm^−1^ to 400 cm^−1^. Each sample was measured three times for mean value calculation. 

#### 2.3.3. Thermal Stability

The thermal stabilities of the CNF, RWSC, and all-cellulose nanocomposite films were measured using a thermal gravimetric analyzer (TGA) (Shimadzu TGA-50) with a platinum–rhodium alloy crucible and N_2_ protection. The test sample weight was 10–13 mg, and the temperature range was 30–650 °C.

#### 2.3.4. Mechanical Properties

The mechanical properties of the films were characterized by a universal testing machine (Xinsansi CMT-2502). Test specimens were cut into pieces that were 50 × 20 mm in length and width at tensile rates of 0.05 mm/min and 5.0 mm/min, respectively. The yield strength, tensile strength, and Young’s modulus were calculated. Each sample was measured three times. Significant differences among the specimens were analyzed by multiple comparisons, and the effects of the concentration of CNF on the film’s mechanical properties were determined.

## 3. Results and Discussion

### 3.1. Morphology of CNFs, RWSC, and All-Cellulose Nanocomposites

**Figure 2:**
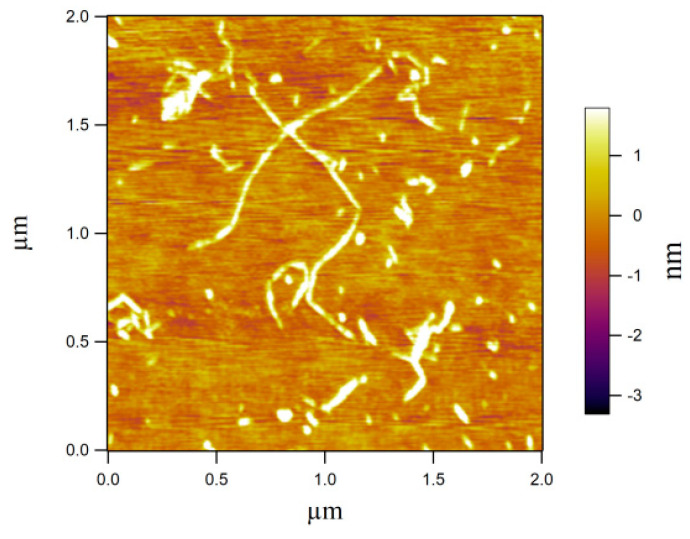
Dimension of CNFs determined using the AFM.

An image of the CNFs obtained using the AFM is shown in Figure 2. Aggregates of individual nanocellulose were the main problem in observing the CNF morphology. After the CNF suspension was diluted and dispersed with ultrasonic vibration, the AFM image showed that the CNFs were 20−50 nm in diameter and 50 nm−2 μm in length. 

**Figure 3:**
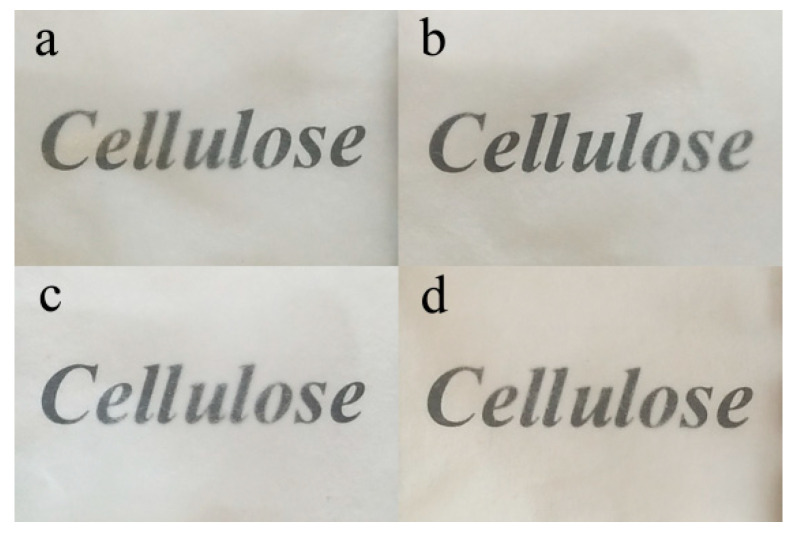
Photograph of specimens prepared by AM: (**a**) RWSC; (**b**) 0.1% CNFs; (**c**) 1.5% CNFs; (**d**) 3.0% CNFs.

**Figure 4:**
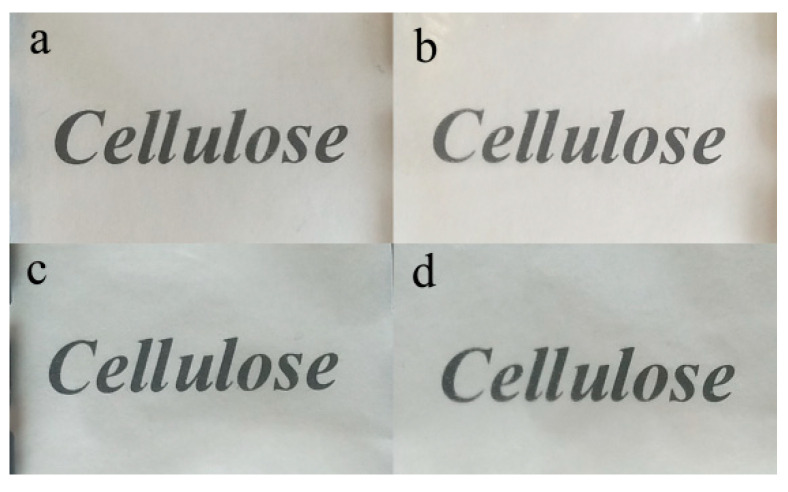
Photograph of specimens prepared by EM: (**a**) RWSC; (**b**) 0.1% CNF; (**c**) 1.5% CNFs; (**d**) 3.0% CNFs.

A film’s optical transparency can be used to evaluate the miscibility of its composite elements [37]. Figure 3 and Figure 4 show the optical photographs of the RWSC and all-cellulose nanocomposite films, which presented good visible-light transparency. Figure 3 shows the Composite-A films with the cellulose extracted by AM. Figure 4 shows the Composite-E films with the cellulose prepared by EM. The letters forming “Cellulose” can be observed clearly in the films. However, under the different cellulose matrices, there were different degrees of clarity among the different samples. Under Composite-A, the letters became blurred with an increase in the CNF concentration, especially for the 1.5% and 3.0% CNFs, as shown in Figure 3. Excluding shrinkage of the films, aggregates of CNFs should have been the main reason, because the blur could have been induced by a phase separation that caused light scattering at the interface of the cellulose matrix and the CNF aggregates [22]. For the Composite-E films, the letters were almost equally clear, as shown in Figure 4. This indicated that the dispersibility of the CNFs was good in the cellulose matrix. As a result, the miscibility of the cellulose matrix and CNFs in Composite-E was better than that in Composite-A. 

Meanwhile, the letters beneath the RWSC film shown in Figure 4 were clearer than those shown in Figure 3, which indicated that solubility of the cellulose prepared by EM was better than that prepared by AM. On the one hand, decolorization and removal of fats and waxes could have been incomplete in the acid–alkali extracting process. On the other hand, the solubility of different WSCs could have been different, even under the same conditions of solution preparation. 

**Figure 5:**
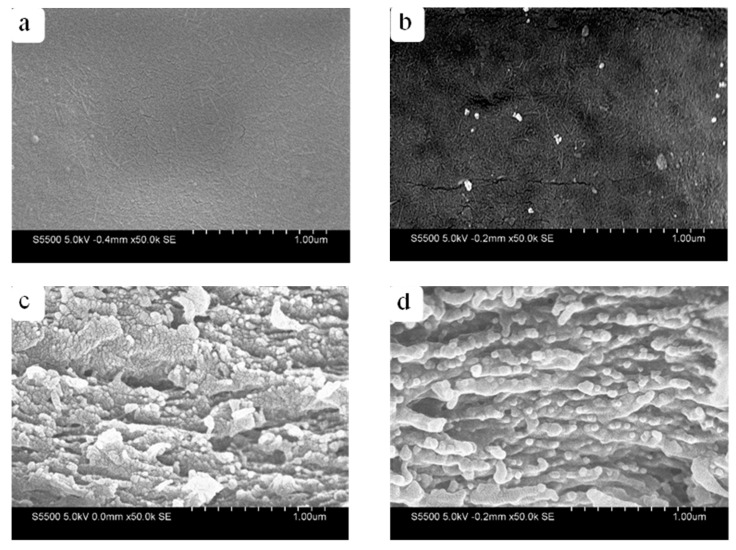
Morphologies of RWSCs prepared by AM and Composite-A films; (**a**,**b**) surface; (**c**,**d**) cross-section.

Figure 5 shows the SEM images of the RWSC and Composite-A films with 3.0% CNFs. Figure 5a,b indicate a similar surface morphology for the two films, except that Figure 5b shows some solid particles, which could have been the undissolved aggregated CNFs on the surface of Composite-A. When observing the cross-sectional images in Figure 5c,d, the RWSC and Composite-A were also in a layered structure, as reported in the literature [38,39]. However, more uniform line fibers were observed in the cross-section of Composite-A, as compared to RWSC. It could be that the ordered arrangement of line fibers made Composite-A more compact. 

**Figure 6:**
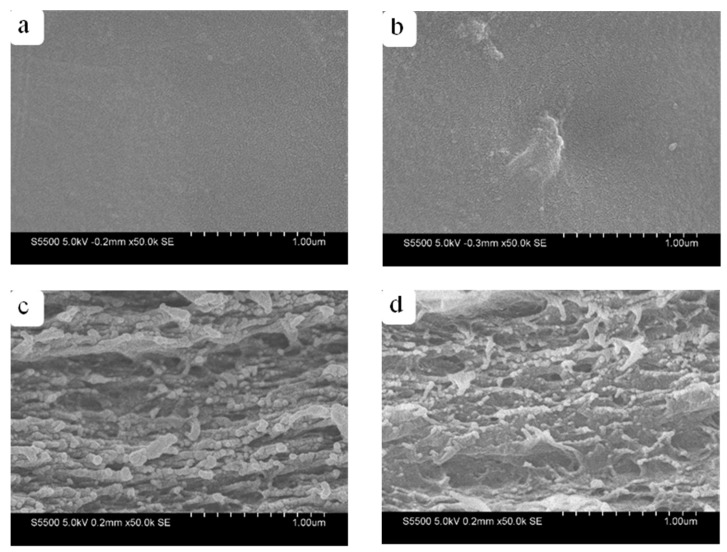
Morphologies of RWSCs prepared by EM and Composite-E films: (**a**,**b**) surface; (**c**,**d**) cross-section.

Figure 6 shows the SEM images of the RWSC and Composite-E films with 3.0% CNFs. Figure 6a,b show that the surface morphologies of two films were similar. Again, the undissolved block-shaped spots can be observed. This could have been the unevenly dispersed aggregated CNFs. The cross-sectional images of Composite-E (Figure 6c,d) also revealed a layered structure similar to that of Composite-A. However, the quantity and diameter of the line fibers in the two cross-sections were visually different between the RWSC and Composite-E films. Overall, the SEM images indicated that the film morphologies were affected by the cellulose extraction methods. 

### 3.2. Structural Characteristics of CNF, RWSC and All−Cellulose Nanocomposites 

**Figure 7:**
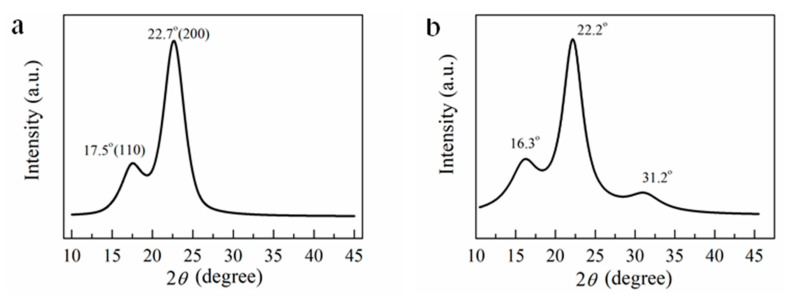

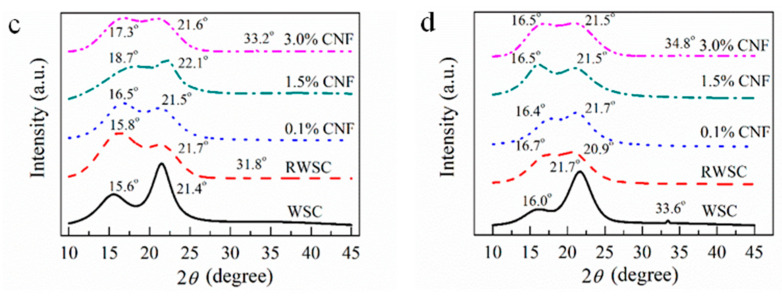
XRD curves of CNFs, WSC, RWSC, and all-cellulose nanocomposites: (**a**) CNFs; (**b**) wheat straw powder; (**c**) WSC prepared by AM; (**d**) WSC prepared by EM.

XRD is a common method to investigate the structures of cellulose, cellulose nanostructures, and cellulose composites [40,41]. Figure 7 shows the XRD patterns of the CNFs, wheat straw, WSC, RWSC, and all-cellulose nanocomposite films. 

Figure 7a shows the diffraction pattern of CNFs isolated from the wheat straw. The crystalline peaks were observed at 17.5° (10 $\overset{-}{1}$) and 22.7° (200), which typically represent the Cellulose I allomorph. However, the CNF XRD pattern did not show the third peak that appeared on the WSC XRD pattern at 31.2° (Figure 7b), which represented the crystal plane (300) in Cellulose I. The calculated CNF crystallinity was 57.5%, which was higher than the wheat straw crystallinity (42.2%), and also significantly higher than that reported by Alemdar [42]. 

As shown in Figure 7c, the main diffraction peak in the AM-extracted WSC arose at around 21.4°, and the secondary peak was around 15.6°. Compared with the wheat straw and CNF diffraction peaks at 22.2° and 22.7° (200), respectively, the main diffraction peak location in the AM-extracted WSC was shifted, indicating that Cellulose II formed. Mercerization could have been the main reason for this transformation [43]. This phenomenon has been reported in the literature [44,45,46]. The concentration of alkali also could have been a key factor [47,48]. Furthermore, it was found that the WSC was a semicrystalline Cellulose I allomorph, and the diffraction peaks of the crystal planes (101) and (10 $\overset{-}{1}$) merged together [49]. This indicated that preparing cellulose using different extracting methods can lead to different crystal structures.

As shown in Figure 7d, the main and secondary diffraction peaks in the EM−extracted WSC appeared at around 21.7° and 16.0°, respectively. These peak locations were close to those exhibited in the XRD curve of the AM-extracted WSC, indicating a similar shifting trend from Cellulose I to Cellulose II. In particular, the secondary peak 16.0° (110) was significantly weakened and flattened. Therefore, although WSC was produced using the different extraction methods, such as the ultrasonic treatment only used in the AM extraction process, the resulting WSC crystalline types were similar. However, the two different extraction methods resulted in two different crystallinities, with 68.7% for AM and 50.7% for EM (Table 1). The higher crystallinity of the WSC prepared by AM indicated that the alkaline and ultrasonic treatments helped remove lignin and hemicellulose more efficiently.

The RWSC films produced from the WSC solutions using the DMAc/LiCl solvent possessed crystal structures from regenerated cellulose. Figure 7c shows the XRD curve of the RWSC film from the AM-extracted WSC. The diffraction peaks were 15.8° and 21.7°. The XRD curve for the RWSC film from the EM-extracted WSC is plotted in Figure 7d, showing a secondary diffraction peak at 16.7° and a main diffraction peak at 20.9°. The diffraction peaks at 2*θ* = 15.8° and 16.7° corresponded to the crystal plane (110) from Cellulose I, and the diffraction peaks at 2*θ* = 21.7° and 20.9° corresponded to the crystal plane (200) from Cellulose II. After the WSC was dissolved and regenerated, the diffraction peak intensity decreased, and the peak width increased. Interestingly, in Figure 7c, the secondary diffraction peak at 15.8° shows the highest diffraction intensity for the RWSC from AM, in contrast to the RWSC from EM (Figure 7d). Therefore, in the RWSC and WSC, the cellulose I crystal structure and cellulose II coexisted. These XRD data indicated that the cellulose crystal type did not change in the dissolution and regeneration process, but the intensity of diffraction peak was determined by the method to prepare the WSC. This result was different from that reported in another reference that the regenerated cellulose had a Cellulose II structure that resulted from the change of Cellulose I to Cellulose II, after the cellulose was dissolved in the LiCl/DMAc solution [25].

Figure 7c,d also show the XRD curves of the all-cellulose nanocomposite films regenerated from the WSC/CNF composites. The coexistence of Cellulose I and Cellulose II was still observed, in addition to two crystalline changes. As one change, the diffraction intensity of the main and secondary peaks tended to be equal as the amount of CNF increased. This indicated that the added CNF did influence the cellulose’s molecular arrangement in the WSC/CNF composites [50]. As another change, after adding CNF, there were fluctuations in the diffraction intensities in the composites with different CNF percentages. This could have resulted from the treatment methods in the extraction process of the cellulose, such as the ultrasonic treatment. Cell walls and crystal structures were broken by ultrasonic cavitation with strong vibration [51]. So, more seriously broken Cellulose II structures could have been produced in the AM process that used the ultrasonic treatment. In dissolving, the intruded solution could reduce the Cellulose II. The cellulose crystal types include Type I, Type II, Type III, and Type IV, but which one represents the best mechanical strength is still inconclusive, and possibly is Type I [24]. This is a complex problem mainly related to the source of the cellulose materials, processing methods, etc. 

The mechanical and thermal properties of the WSC, RWSC and composite films were largely determined by crystallinity. The crystallinity of the WSC, RWSC, and all-cellulose nanocomposites are listed in Table 1. The crystallinity of the RWSC was lower than that of the WSC, and exhibited a consistent result with that reported in the literature [25]. It was expected that the addition of CNFs could improve the RWSC’s crystallinity, but the result was contrary to that for Composite-A. One reason was that the aggregation of CNFs induced by the increasing CNF content could cause the decrease in crystallinity [52]. Another reason was that the addition of CNFs could be attributed to the Cellulose I conversion to Cellulose II when dissolving to form an amorphous structure [53]. In contrast, the crystallinity of Composite-E increased with the increase in CNF content, especially at the concentrations of 1.5% and 3.0%. Obviously, here the cellulose extraction methods became a critical factor in determining the crystallinities of the all-cellulose nanocomposite films. 

**Figure 8:**
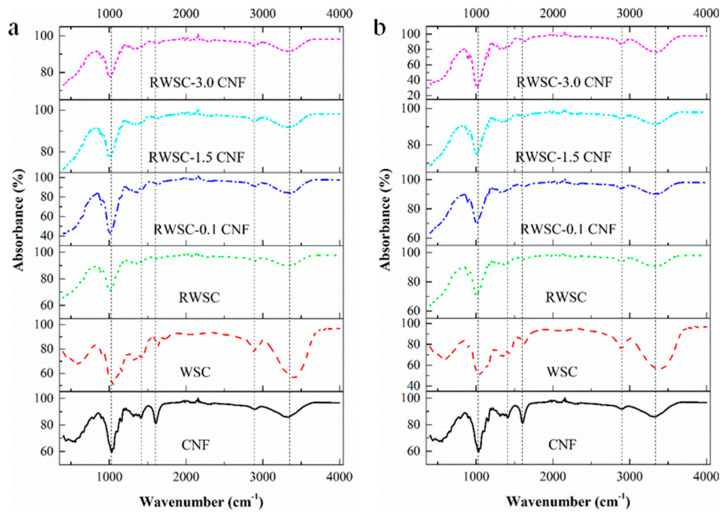
FTIR curves of WSC, RWSC, and all-cellulose nanocomposites: (**a**) WSC prepared by AM; (**b**) WSC prepared by EM.

Figure 8 displays the FT-IR spectra of the CNFs, WSC, RWSC, and composite films. The strong hydrogen-bonded O-H stretching vibration is indicated in the 3408–3273 cm^−1^ region for the RWSC and Composite-A, as shown in Figure 8a; and the 3388–3240 cm^−1^ region for the RWSC and Composite-E, as shown in Figure 8b. For the two WSC cellulose samples, the maximum absorbance of the O-H vibration were intramolecular hydrogen bonds in Cellulose I, appearing at 3408 and 3388 cm^−1^, respectively [54,55]. After the cellulose was dissolved and the CNFs were added, the wave numbers of the hydrogen-bond O-H groups were shifted toward a low frequency. In the frequency range of 3230–3570 cm^−1^, intermolecular and intramolecular hydrogen bonds in the valence vibration of the H-bonded O-H groups both existed [54,55]. This indicated that the strengths of the hydrogen bonds were balanced by dissolving process. Because of this, Cellulose I did not change within this frequency range [56].

The frequency range of 1162–1056 cm^−1^ indicates the asymmetric and symmetric C-O-C stretching vibration. There were absorbance peaks at 1162 and 1056 cm^−1^ for the WSC prepared by AM; and there were absorbance peaks at 1160, 1110, and 1058 cm^−1^ for the WSC prepared by EM. After the two celluloses were dissolved and CNF was added to prepare the RWSC and Composite-E, the FTIR absorbance decreased and shifted. The peaks at 1162 and 1160 cm^−1^ both were shifted to 1157 cm^−1^, and 1056 cm^−1^ was shifted to 1078 cm^−1^. The peak at 1058 cm^−1^ was no longer found. Alkali treatment in natural cellulose fiber can convert Cellulose I to Cellulose II, which has been reported previously [57,58]. Due to the alkali treatment used in the both cellulose-extraction methods, the absorbance peaks at 1162, 1160, 1058, and 1056 cm^−1^ indicated the presence of Cellulose II, which was shifted from the original Cellulose I. The absorbance peaks at 1162 and 1160 cm^−1^ both came from 1163 cm^−1^ in Cellulose I, and the change in intensity at 1056 cm^−1^ also represented the conversion of Cellulose I to Cellulose II [57]. Considering all these facts, the results showed a tendency toward a crystal structure change from Cellulose I to Cellulose II. With this conversion, some researchers believed that the crystallinity also decreased [58].

The 1430–1420 cm^−1^ bands belong to the CH_2_ symmetric bending mode. If the cellulose had a plentiful amount of crystal Cellulose I, this band shifted to 1430 cm^−1^. However, 1420 cm^−1^ meant the existence of Cellulose II and amorphous cellulose [59,60], and 1425 cm^−1^ suggested a presence of Cellulose III [61]. In Figure 8, the band at 1429 cm^−1^ appears in the both AM-extracted and EM-extracted WSCs. In Figure 8a (WSC prepared by AM), this absorption band is shifted from 1429 cm^−1^ to 1425 cm^−1^ after the cellulose regeneration and CNF addition, indicating a structural transformation from Cellulose I to Cellulose III. In Figure 8b (WSC prepared by EM), this absorption band is shifted from 1429 cm^−1^ to 1421 cm^−1^, indicating a structural change from Cellulose I to Cellulose II. If the bands at 1430 and 1111 cm^−1^ were not observed, it implied that crystalline Cellulose I was practically nonexistent in the cellulose sample [59]. So, it could be understood that the crystal structure of all the regenerated cellulose films produced in this study was changed from Cellulose I. 

The absorbance bands at 1263 and 1198 cm^−1^ appeared for the RWSC and all-cellulose nanocomposite films. The band at 1263 cm^−1^ was assigned to C-O-H bending in the plane at C-2 or C-3 [60]. It represented Cellulose I and Cellulose II. The increase in the absorbing intensity indicated that the crystal structure shifted from Cellulose I to Cellulose II [55]. Little has been reported about the band at 1198 cm^−1^ in previously published works. At 1200–1202 cm^−1^, researchers considered that the absorbance was caused by C-O-H bending in the plane at C-6 [60]. When the peak shifted from 1202 cm^−1^ to 1200 cm^−1^, the crystal structure transferred from Cellulose I to Cellulose II [55]. Based on this, it could be illustrated that the absorbance at 1198 cm^−1^ resulted from the shift of 1202 cm^−1^ toward Cellulose II.

The band at 1635 cm^−1^ has been known as bound water [56], and it is weaker when the crystallinity is greater. It was interesting to observe that this 1635 cm^−1^ absorbance for all the RWSC and composites films increased, trending toward a crystallinity decrease in these films [60]. That could have been a result of the elevated vibration of bound water molecules in the amorphous area. Other researchers reported that the absorbance peak was at 1610 cm^−1^ after the cellulose was oxidated by TEMPO [62]. When the TEMPO-oxidated CNF was added into the WSC for dissolving, this absorbance peak was not observed in all the all-cellulose nanocomposite samples. A CNF concentration that was too low might have been reason for this.

### 3.3. Thermal Performance Analysis

**Figure 9:**
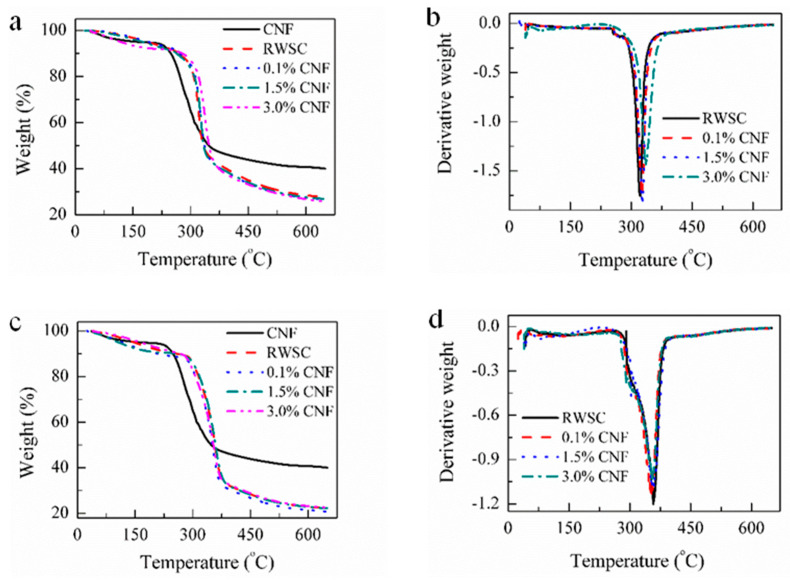
TGA and DTG curves of the CNFs, RWSC, and all-cellulose nanocomposite: (**a**,**b**) WSC prepared by AM; (**c**,**d**) WSC prepared by EM.

To investigate the influence of CNFs on the thermal performances of the all-cellulose nanocomposites, TGA measurements of CNFs, RWSC, and all-cellulose nanocomposites were taken. Figure 9a,b show the TGA and DTA curves of the AM-derived RWSC and all-cellulose nanocomposites. A one-stage degradation model is presented in all the TGA curves during mass loss, with a differentiation among CNFs, RWSC, and Composite-A. The start temperature of the major mass loss for the CNFs was about 230 °C, which was significantly lower than the start temperatures of the major mass loss at around 290 °C for RWSC and Composite-A with 0.1%, 1.5%, and 3.0% CNF. It was also observed that the addition of CNFs increased the mass-loss rate of the nanocomposites. This led to a low temperature for quickly reaching the maximum mass loss rate; i.e., 321, 326, 327, and 336 °C, as shown in the DTG curves. 

The TGA and DTA curves of the EM-derived RWSC and all-cellulose nanocomposites are shown in Figure 9c,d. Compared to the TGA and DTG curves in Figure 9a,b, the start temperatures of major mass loss for the RWSC and composite films were different. The start temperature of the major mass loss was in the range of 272–292 °C for the RWSC and Composite-E with 0.1%, 1.5%, and 3.0% CNF, similar to the 290 °C for the AM-derived RWSC and Composite-A. However, the temperatures corresponding to the maximum mass loss rate were 360, 353, 358, and 355 °C, respectively. Therefore, Composite-E showed a better thermal stability in comparison with Composite-A. 

### 3.4. Mechanical Properties of the All-Cellulose Nanocomposites

The mechanical properties of the all-cellulose nanocomposites were tested. The tensile strength, yield strength, and Young’s modules of Composite-A and Composite-E at tensile speeds of 0.05 and 5 mm/min are listed in Table 2. 

When the added CNF concentrations were increased, almost all of the tensile strengths of the all-cellulose nanocomposites also were increased. This tendency was consistent with the results given in a previous report [26,63]. When viewing the tensile strengths of the nanocomposite films with different CNF concentrations, the Composite-E films were better than the Composite-A films. This result could be verified by the analyses of the crystal type and crystallinity in the XRD and FT-TR data. In Composite-E, the Cellulose I content was higher than the content of Cellulose II, and the crystallinity was also higher than that of Composite-A. A richer Cellulose I structure and a higher cellulose crystallinity tended to result in stronger mechanical properties.

The tensile speed influenced the test results of the all-cellulose nanocomposites’ mechanical properties. With an increase in the tensile speed, the yield strength and Young’s modulus increased correspondingly for Composite-A. The tensile strength of Composite-A with 0% CNF increased from 29.1 MPa at 0.05 mm/min to 39.0 MPa at 5 mm/min. For Composite-E, the yield strength, tensile strength, and Young’s modulus all increased with a tensile speed increase. This indicated that the cellulose matrix film materials prepared by EM were more sensitive to the strain rate; that is, the higher the tensile speed, the higher the tensile strength. Depending on the influence of content, aggregation, and phase separation of the CNF [64], the increments in tensile strengths, yield strengths, and Young’s moduli of the all-cellulose nanocomposite films were not as significant as for the other materials. 

To determine why there was no significant tendency in the experimental data, a statistical analysis of the experimental data was carried out. Table 3 shows the results of the multiple comparisons for tensile strength. For Composite-A, there was a significant difference between the RWSC and Composite-A with different CNF contents at a tensile rate of 0.05 mm/min, but no significant difference with the addition of CNF at 5 mm/min. For Composite-E, there was a significant difference between the RWSC and Composite-E with 3.0% CNF for both tensile speeds; and a significant difference between Composite-E with 3.0% CNF and Composite-E with 0.1% and 1.5% CNF contents at 5 mm/min. The statistical-analysis results indicated that the CNF content was a key factor in the significant differences in mechanical properties between the all-cellulose nanocomposites and the RWSC. When the CNF content reached 3.0%, the tensile strengths of the all-cellulose nanocomposite films were significantly enhanced. Nevertheless, the dispersion of CNF in the LiCl/DMAc solvent system could be also an issue that affected the composite films’ mechanical properties.

## 4. Conclusions

The CNF extracted from wheat straw had a Cellulose I structure; the diameter and length were 20–50 nm and 50 nm–2 μm, respectively; and the thermal-decomposition temperature was 278 °C. The WSC prepared using AM and EM both had coexisting crystal structures of Cellulose I and Cellulose II. The crystallinity of the AM-extracted WSC was 68.7%, higher than that of the EM-extracted WSC, which could mean that the ultrasonic treatment caused more crystal structure to be exposed in the AM.

The RWSC and all-cellulose nanocomposite films prepared with CNF and WSC both presented high optical transparencies. The degree of clearness of the printed letters under the films was influenced by the WSC. It was almost equally clear under Composite-E, but it became blurred with CNF content under Composite-A, which indicated that the miscibility of the WSC matrix and CNFs in Composite-E was better than in Composite-A. All of the films showed a layered cross-section, and the all-cellulose nanocomposite films exhibited more uniform line fibers, which made them more compact.

The thermal-decomposition temperature of the films was around 290 °C, but the maximum mass loss temperature varied with the WSC. The temperature of Composite-E was higher than that of Composite-A, and showed a better thermal stability. The removal rate of organic substances in the cellulose could have been the main influence on the thermal stability. 

All of films presented the coexisting crystal structure of Cellulose I and Cellulose II, and the diffraction intensity of two peaks in the XRD tended to be equal with a CNF increase. The Cellulose II structure was more significantly detected in the AM-extracted WSC, which should have been caused by the ultrasonic treatment. A transition of Cellulose I to Cellulose II was found in the FT-IR spectra with a CNF addition, and the shifting processes were affected by the preparation methods of the WSC. The higher Cellulose I content observed in the shifting character of the absorption peaks in Composite-E could have been caused by bound water, which caused the crystallinity to decrease in the RWSC and all-cellulose nanocomposites 

The addition of CNF improved the tensile strengths of the composite films, with a higher value for Composite-E than Composite-A due to the higher crystallinity and Cellulose I content. With a CNF increase, there was a strain-rate response in the yield strength, Young’s modulus, and tensile strength of composite-E, and also in the yield strength and Young’s modulus of Composite-A, but not in the tensile strength of Composite-A. Moreover, the multiple-comparisons analysis indicated that the tensile strengths of the all-cellulose nanocomposite films both increased when CNF concentration reached 3.0%. 

The added value of wheat straw can be improved by extracting cellulose, CNF, and preparing all-cellulose nanocomposite film. The above results are also expected to provide practical experience and enrich the experimental data for deep processing of wheat straw and agricultural residues. At the same time, the all-cellulose composite films we prepared and charactered also showed potential for application as a biodegradable film to substitute for fossil-fuel-based films.

## Figures and Tables

**Table 1:** Crystallinity index (CrI%) of WSC, RWSC, and all-cellulose nanocomposite.

Extraction Method	WSC	RWSC	All-Cellulose Nanocomposite		
			0.1%	1.5%	3.0%
Prepared by AM	68.7	55.5	47.9	42.9	46.9
Prepared by EM	50.7	44.3	40.8	58.9	50.9

**Table 2:** All-cellulose nanocomposite mechanical properties of two WSCs prepared by AM and EM.

WSC	Strain Rate (mm/min)	CNFs (%)	Yield Strength (MPa)	Tensile Strength (MPa)	Young’s Modulus (GPa)
Prepared by AM	0.05	0	6.5 ± 2.1	29.1 ± 6.6	0.32 ± 0.07
		0.1	11.0 ± 2.1	46.1 ± 15.0	0.55 ± 0.08
		1.5	21.3 ± 2.1	37.3 ± 4.2	1.07 ± 0.14
		3.0	15.4 ± 2.1	53.6 ± 13.0	0.77 ± 0.18
	5	0	22.6 ± 4.5	39.0 ± 6.3	1.13 ± 0.23
		0.1	26.6 ± 0.14	40.5 ± 7.9	1.41 ± 0.07
		1.5	29.2 ± 0.19	39.7 ± 0.01	1.40 ± 0.07
		3.0	22.6 ± 0.08	41.4 ± 0.1	1.13 ± 0.00
Prepared by EM	0.05	0	11.1 ± 2.1	38.0 ± 1.8	0.56 ± 0.10
		0.1	13.1 ± 2.1	41.5 ± 12.0	0.66 ± 0.13
		1.5	19 ± 2.1	56.0 ± 3.8	0.95 ± 0.24
		3.0	12.3 ± 2.1	65.1 ± 2.2	0.61 ± 0.27
	5	0	24.0 ± 4.2	42.0 ± 4.3	1.20 ± 0.21
		0.1	24.1 ± 2.4	50.1 ± 1.4	1.21 ± 0.12
		1.5	20.8 ± 1.3	52.9 ± 2.9	1.04 ± 0.06
		3.0	18.5 ± 3.7	59.6 ± 9.1	0.93 ± 0.18

**Table 3:** Influence of CNF on all-cellulose nanocomposite mechanical properties.

WSC	Strain Rate (mm/min)	CNF Content	0.0%	0.1%	1.5%	3.0%
Prepared by AM	0.05	3.0%	20 **	5	7	
		1.5%	13 *	2		
		0.1%	15 **			
		0.0%				
	5	3.0%	5	7	4	
		1.5%	2	4		
		0.1%	2			
		0.0%				
Prepared by EM	0.05	3.0%	28 *	19	11	
		1.5%	17	9		
		0.1%	8			
		0.0%				
	5	3.0%	17 **	19 **	24 **	
		1.5%	7	5		
		0.1%	2			
		0.0%				

Note: ** very significant differences; * significant differences.

## Data Availability

The data presented in this study are available on request from the corresponding author.
